# Continuing Education in Digital Skills for Healthcare Professionals — Mapping of the Current Situation in EU Member States

**DOI:** 10.34172/ijhpm.8309

**Published:** 2024-06-24

**Authors:** Anu-Marja Kaihlanen, Lotta Virtanen, Emma Kainiemi, Virpi Sulosaari, Tarja Heponiemi

**Affiliations:** ^1^Department of Public Health and Welfare, Finnish Institute for Health and Welfare, Helsinki, Finland.; ^2^Faculty of Health and Well-being, Turku University of Applied Sciences, Master School, Turku, Finland.

**Keywords:** Continuing Education, Training, Digital Competence, Healthcare Professionals, European Union

## Abstract

The rapid advancement of technology in healthcare is creating new competency requirements for professionals, such as skills for data management and the adoption of new technologies, understanding the effect of digitalisation on clinical processes, and evaluating clinical safety and ethics within the context of digitalisation. These requirements call for improved educational curricula and ongoing continuing education in digital skills. This study, as part of the Digital Skills Training for Health Care Professionals in Oncology (DigiCanTrain) project, aims to map and describe the existing continuing education in digital skills for healthcare professionals (HCPs) in European Union (EU) Member States. Using a mapping study methodology, data was collected from experts in 25 EU countries through surveys and from online sources. Qualitative content analysis was used for categorising the data. The results show variations between countries in policy strategies, training organisation, and funding mechanisms. Educational institutions, employers, third parties, and national/regional authorities were found to be the main organisers of the digital skills training. Comprehensive accreditation systems seemed to be scarce, and practices also varied between countries. The study highlights the importance of adopting a systematic approach to enhancing continuous professional development in digital skills, which would ensure that professionals have equitable access to education, resulting in consistent, quality patient care across countries and regions. The findings offer valuable insights for policymakers, educators, healthcare institutions, and professionals.

## Background

 Constantly evolving technology and digital solutions are transforming healthcare and creating novel competence requirements for the professionals. Despite the current use and positive effects of eHealth technology, many healthcare professionals (HCPs) may feel insufficiently trained to cope with the digital revolution. The barriers to technology adoption include the absence of a set of knowledge and skills among HCPs regarding the use of eHealth technology.^1^ Moreover, it has been acknowledged that the curricula for HCPs are insufficient to respond to the competence requirements created by the expanding digitalisation.^1-3^ Therefore, the need to enhance the educational curricula for HCPs and utilise ongoing professional development initiatives for digital skills training has been widely recognised.^1,3-5^

 Although evidence of the importance of digital skills training has grown substantially in recent years,^6^ in many countries, progress in integrating digital health content into continuous professional development has been substantially slow.^3^ Significant efforts have been put into defining essential digital skills. For example, the International Medical Informatics Association recommends core competences for HCPs in digitalisation across six domains. These encompass essential knowledge and skills, such as data management, understanding the impact of digitalisation on clinical processes, proficiency in adopting new technologies such as blockchain, and evaluating clinical safety and ethics within the context of digitalisation.^5^ However, the implementation of core competences has been found to be lacking, and there is a need for a deeper understanding of how these skills should be developed and integrated into education.^7^ The formulation of educational programme content for HCPs is commonly influenced by both higher education establishments and professional associations, based on minimum regulatory requirements.^8^ In some countries governmental initiatives have been launched to augment the involvement of alternate stakeholders, such as healthcare organisations, in this process.^9^

 To enhance the transparency, comparability, and efficiency of continuing education programmes and to support the professional growth of individuals, the significance of study certificates and study points has been underscored.^10^ The European Union (EU) has mandated the utilisation of the European Credit Transfer and Accumulation System (ECTS) credits in higher education institutions and is progressively encouraging their adoption in courses that lead to regulated professional qualifications.^4^

 This study aims to map and shed light on how the continuing education on digital skills for HCPs is currently arranged and implemented in EU Member States. Doing so will contribute to the discussion about the importance of the continuous development of digital skills of HCPs. Moreover, mapping the current situation will provide indicative comparative information that can be utilised for developing continuing education models for digitalised healthcare work in Europe.

## Methods

 A mapping study^11^ was conducted to identify existing opportunities for continuing education in digital skills for HCPs in EU countries. Instead of mapping existing literature or documents, an expert-based approach was used. This was because information on the specific practices in different countries was not expected to be publicly available, and searching for it would have been unfeasible due to the variety of languages involved.

 The study was conducted as a part of the Digital Skills Training for Health Care Professionals in Oncology (DigiCanTrain) project.^12^ The project has received funding from the EU, EU4Health Programme 2021–2027 as part of Europe’s Beating Cancer Plan under Grant Agreement no. 101101253. The views and opinions expressed are however those of the authors only and do not necessarily reflect those of the EU or the Health and Digital Executive Agency (HaDEA). Neither the EU nor the granting authority can be held responsible for them. While the DigiCanTrain project is specifically focused on the field of oncology, the emphasis of this study was on the continuing education opportunities available to HCPs in different settings.

###  Recruitment of Experts and Data Collection

 An online survey, Brief Survey on Continuing Education of Healthcare Professionals’ Digital Skills in the EU Member States, was conducted among a group of international experts with knowledge of the topic. The survey consisted of three open-ended questions: (1) How is the development of digital skills of HCPs organised in your country, (2) which stakeholders are involved in organising the education/training and is there a main responsible national authority coordinating the implementation, and (3) is the continuing education/training on digital skills accredited or certificated, or mainly in-house training without any official credits (continuing medical education, ECTS)?

 The questions underwent initial piloting with two experts, and their feedback was used to refine the wording of the questions.

 Multiple experts were contacted in all 27 EU Member States who were identified through our research group’s existing networks. An expert was defined as an individual with expertise in the current subject area, either through their position/role, workplace, or research experience. Contact details were found by searching relevant national ministries, educational institutions, European-level organisations, and research articles. The individuals were contacted via email, inviting them to participate in the survey. The email contained information about the aims of the study and the DigiCanTrain project, and the privacy notice of the survey was provided. Participation was voluntary, and the experts’ identities were kept confidential. The contacted individuals had the option to respond to the survey themselves or suggest potential experts from their respective countries to whom the survey could be forwarded.

 The survey was answered in a Word document attached to the invitation email, and the participants were instructed to return it to the research group via email. The survey questions were in English, and the respondents provided the answers in English or, in a few cases, their native language. Native-language responses were translated into English using artificial intelligence (AI) assistance.

 Additionally, as it was not possible to contact experts from all EU countries, the research was extended to seek answers to the survey questions from policy documents, research articles, and other information available in English from relevant websites. Searches were conducted in national languages using AI to translate our search terms, and vice versa, foreign-language texts were translated into English as needed.

 The data covered information from all EU countries except Bulgaria and Luxembourg, for which no expert or online information could be located. Thus, the data analysis is based on information from 25 EU Member States, with information from 20 of these countries being provided mainly by experts (n = 24) and information from five countries being derived from only online sources (presented in Table S1 in Supplementary file 1). The experts were professionals working in the fields of digital health, healthcare, education, or national positions.

###  Data Analysis and Synthesis

 The anonymised survey answers and data collected from online sources were analysed using a qualitative content analysis.^13^ The experts’ responses to the questions were brief, averaging from one to three sentences, in which they succinctly described the main features of the implementation of continuing education in digital skills for HCPs in their country. Two authors (A-MK and LV) thoroughly reviewed the data and extracted and condensed all the responses into descriptions that closely reflected the original expressions. Subcategories were formed by grouping descriptions (condensed expressions) with similarities, and these subcategories were further organised into three main categories (themes) based on the similarity of content. A descriptive synthesis of the information obtained from each category is presented in the following section. Supplementary file 2 presents all condensed expressions and categories by country.

## Results

 The following paragraphs summarise the implementation of continuing education for HCPs in digital skills in different EU countries. The content analysis revealed three main themes: (1) policy/strategic level incentives and initiatives, (2) organisation of training, and (3) funding (See coding tree in Supplementary file 1).

###  Policy/Strategic Level Incentives and Initiatives 

 The descriptions of the experts about the situation around policy and strategic-level incentives and initiatives behind the continuing education were roughly divided into two perspectives: in some countries, continuing education was clearly driven by national strategies and the authorities, whereas in others a lack of national coordination and a systematic approach in continuing education were reported.

####  National Coordination of Continuing Education

 Based on the descriptions, national-level coordination of digital skills development for HCPs varies between countries and is driven by tailored strategies, governmental authorities, and collaboration among stakeholders.

 Many countries demonstrated a commitment to enhancing the digital skills of HCPs through national strategies. These strategies cover various aspects, including specialised training, interdisciplinary workforce development, and financial incentives. For example, Slovenia and Spain have established strategies that aim for workforce specialisation, Croatia has emphasised lifelong learning through a strategy involving universities and trade unions in co-designing and implementing educational programmes, and Croatia has planned financial incentives to foster the provision of continuing education in organisations.

 The role of governmental organisations, such as national eHealth agencies and those responsible for continuous professional development, were described. The role of the governmental authorities varies but primarily they oversee continuing education (including digital skills training) and ensure compliance with national standards. As an example, in France the National Agency for Continuous Professional Development manages and oversee training, but the Spanish Ministry of Health primarily focuses on guideline formulation, leaving the coordination of training to other parties. In Hungary and the Czech Republic the supervision of healthcare and education development is centralised under specific ministries, whereas the Netherlands and Poland have adopted a more collaborative approach involving multiple stakeholders (eg, municipalities, human resource professionals, entrepreneurs, and information technology departments of organisations) in these actions.

 To ensure that professionals remain up to date with the latest knowledge and practices, in some countries (eg, Austria, Belgium, and Slovenia), continuing education in digital skills is mandated to maintain a professional license. Austria, for example, utilises a point system to track and ensure that HCPs meet their continuous education requirements. Similarly, Slovenia requires physicians to accumulate points through diverse continuing medical education activities for license renewal, whereas Greece mandates certification in basic digital skills for nurses working in the public sector. In the Netherlands, a collaborative initiative “Digivaardig in de zorg” has also promoted a shared understanding of baseline digital competencies and defined a minimum digital skills standards needed by HCPs across sectors.

 In contrast to centralised coordination, in some countries regional-level coordination was also described. Experts from Spain, Italy, and Denmark reported that the coordination of continuing education in digital skills is largely managed at the regional level, meaning that regional authorities and institutions oversee and implement educational initiatives. Both Italy and Denmark involve some coordinating institutes, such as PoliS Lombardia in Italy and Copenhagen Academy for Medical Education and Simulation in Denmark that are responsible for guiding the implementation of training programmes and central coordinating authorities.

####  Lack of National Coordination or Systematic Approach for Continuing Education

 The absence of a national coordination or systematic approach for continuing education in digital skills was described widely. Half of the countries (Belgium, Denmark, Estonia, Finland, Germany, Greece, Ireland, Italy, Lithuania, Malta, Netherlands, Portugal, Romania, and Sweden) lacked comprehensive national regulations or an authority responsible for coordinating the implementation of training on digital skills for HCPs. The experts generally acknowledged the inadequate attention and organisation around the development of digital skills for HCPs and a lack of structures and systems for skills development.

 Some countries (eg, Austria, Ireland, and Italy) reported that broader national strategies to enhance digital skills across the population and public sector existed, however these strategies did not explicitly address digital skills development for HCPs. In the responses from Lithuania and Italy, the consideration of initiating national coordination for training was brought up, indicating a potential shift towards more structured approaches in the future.

###  Organisation of Training

 Four stakeholders—educational institutions, employers, third parties, and national authorities—were identified as organisers of training in digital skills for HCPs. The position of these stakeholders in relation to the responsibility for training varied between countries. Additionally, professionals themselves were often described as being responsible for self-learning and learning on the job. Countries also had different practices for accreditation of continuing education, and these are summarised at the end of this subsection.

####  Educational Institutions as Training Organisers

 Information about educational institutions as training organisers was found from slightly over half of the countries. Most often the information concerned universities, but also university colleges, universities of applied sciences, and postgraduate institutions were mentioned. Primarily it seemed that the organisation of training among educational institutions is centralised to a single or a few organisers, such as in the Czech Republic, Estonia, Finland, Germany, and Latvia. In some countries, such as Austria and Denmark, multiple educational institutions were noted as training organisers.

 In many countries, training offered by educational institutions encompasses shorter study modules, such as three modules of several hours provided by the University of Oulu in Finland. However, a growing number of countries are introducing comprehensive study programmes tailored to obtaining a master’s degree in the field of digital health. As an intermediary option, there is also a weekly three-month international Digital Health Executive Course administered by the National School of Public Health of the NOVA University of Lisbon, responsible for postgraduate teaching in Portugal. Notably, the course has received recognition from the European Institute of Innovation and Technology, EIT-health.

 Regarding the course content, the topics commonly aim at enhancing proficiency in the systematic utilisation of digital tools. These encompass areas such as electronic health records, telemedicine, mHealth, digital clinical guidance, ethical considerations, the application of AI and algorithmic thinking, as well as three-dimensional printing. Notably, in several countries such as Finland, Ireland, and Malta, recent or forthcoming offerings also focus on cultivating leadership skills within the context of the digital health transformation. The methods for delivering training vary, with online courses and hybrid approaches becoming increasingly prevalent.

 Critiques have arisen in certain countries, including Greece and Italy, pointing out deficiencies in the availability of advanced courses that cover a comprehensive range of digital skills within educational institutions. Additionally, despite the existence of courses that cater to different HCPs, some degree of inequality in opportunities for training seems to persist across occupational groups. For instance, in Austria, opportunities for training in digital skills within educational institutions is often restricted to professionals holding a requisite bachelor’s degree at the university level, potentially limiting training opportunities for nurses.

 The available information generally did not indicate how professionals enrol for the courses offered by educational institutions. For example, the question remains as to whether there is employer-provided incentive and whether university courses can be taken during working hours. Nonetheless, experts from a small number of countries, such as Ireland, emphasised that professionals primarily seek out these courses independently.

####  Employers as Training Organisers

 Slightly less than half of our experts mentioned employers as organisers of training. The role of the employer in providing training varied. For example, in the Czech Republic and Romania, training is primarily organised by employers, while in Sweden, employers bear only partial responsibility for its organisation, among other stakeholders. A Finnish expert emphasised that healthcare organisations bear the responsibility for ensuring their personnel’s proficiency in using health information systems and digital devices. Consequently, in Finland, in-house training and support should be provided whenever deemed necessary, although there are no guarantees of practice. In some countries, such as Austria and Germany, only certain employers, such as large hospitals, were reported to provide training, but the information did not specify whether the training venue was centralised or available only to those working under a particular employer. Specific details about the content and frequency of in-house training were generally absent from the information provided.

####  Third Parties as Training Organisers

 Third parties were identified as training organisers in slightly less than half of the countries. For example, in Hungary, Poland, and Slovenia, trade unions have a pivotal role in organising continuing education for HCPs. In Poland, nursing trade unions closely cooperate with the International Council of Nurses in the organisation of the courses. Finland and Italy referred to specialised professional communities, such as the Finnish Society of Telemedicine and eHealth and the Italian Society of Biomedical Informatics, aiming to promote digital skills and offer courses to professionals. Moreover, in Finland, a consulting company was noted, which, in collaboration with wellbeing services counties, arranges an annual training event in digital health. Additionally, in the Netherlands, a private company focuses on training digital coaches from interested HCPs who can serve as peer supporters in their organisations.

####  National or Regional Authorities as Training Organisers

 Only in a few of the countries, were national or regional authorities mentioned as training organisers. In Finland and Lithuania, such national authorities include entities entrusted with overseeing the nationally implemented patient data repository. When this digital system was nationally implemented in Finland, a government agency provided online training materials for new tasks, such as using electronic prescriptions and accessing the national patient data archive. In Lithuania, government-supported training is provided for their national patient data repository periodically and upon request to ensure the proper utilisation of the system, especially during any system updates. While a Finnish expert highlighted that training for national-level implementations might receive employer support and be conducted during work hours, such information was not available for other countries.

 Furthermore, the participating experts mentioned the existence of nationally provided online training, such as in Ireland, where the national health service authority provides an online learning platform offering numerous opportunities for digital education. In the Netherlands, the government supports the Digital Skills in Healthcare initiative, which offers online learning materials and organises events to enhance the digital skills of professionals. Training organised under regional authorities can be found in Denmark, where the Copenhagen Academy for Medical Education and Simulation provides training for practising teleconsultations, combining self-learning online courses with in-person simulation training. Another example comes from Finland where university hospitals have developed a nationally accessible digital service for HCPs, known as HealthVillagePRO, with support from both the government and regional authorities. This digital service includes online coaching to facilitate practice changes related to digitalisation. Additionally, insights from Spain indicated the involvement of both national and regional authorities in organising training.

####  Professionals’ Self-learning and Learning on the Job

 In a few of the studied countries, the emphasis was on the individual professionals’ sole responsibility for their digital skills development. Insights from Malta and Slovenia indicated that the enhancement of digital skills is primarily self-driven and hinges on learning on the job for HCPs. Additionally, in countries with several training organisers, such as in Finland, Ireland, and the Netherlands, professionals can also learn digital skills by themselves through online platforms provided by national authorities or third parties.

####  Accreditation of Continuing Education

 Since opportunities for continuing education in digital skills were notably fragmented across countries, accreditation practices also varied.

 Comprehensive systems of accreditation were scarce, with specific examples found in Austria, France, Portugal, and Romania. In these countries, national frameworks for continuing education points were identified, and professionals participating in courses, webinars, conferences, and other continuing education activities were accredited with nationally recognised points. Moreover, some countries (eg, Austria, Finland, Greece, and Spain) provided opportunities for pursuing degree-based master’s programmes specialising in digital health. Finland also stood out as a country where trade unions can grant specialised competence in health informatics for physicians and nurses.

 Accreditation procedures concerning university-level courses were also mentioned. The ECTS credit system was employed in some countries, including Denmark, Finland, Latvia, Malta, Romania, and Sweden. In some cases, credits were rewarded for a portion of the hours of course participation, as in Finland and Lithuania. In certain countries such as Greece and Ireland, national accreditation systems acknowledge postgraduate training as a distinct level of university accreditation.

 However, accreditation opportunities for in-house training and self-directed learning were limited, although one Swedish expert suggested the possibility of local-level certificates. Ireland appeared to be a unique country where certification could be attained through self-learning on a national digital platform. In contrast, similar courses in the Netherlands did not lead to certification.

 The available information suggests that robust accreditation practices for continuing education in digital skills appear to be currently absent in Belgium, Croatia, the Republic of Cyprus, Germany, Italy, Poland, Slovenia, and Spain. Nevertheless, many of these countries have initiated efforts to further develop the accreditation system.

###  Funding of Training

 Only a few countries provided information on the funding for continuing education in digital skills. Based on the given descriptions, the organisation varied and the funding involved several sources. These included employer contributions (France and Ireland), national authority and publicly supported funding (France and Germany), sponsor-based funding (Austria), and self-financing by professionals themselves (Ireland and the Netherlands). For example, in France, a national agency contributes to the financial management of the continuous professional development system for all HCPs. In addition, public and private employers are obligated to contribute to the funding of continuous professional development actions for their employees.

## Discussion

 The aim of this study was to map how the continuing education in digital skills for HCPs is currently organised and implemented in EU Member States. The mapping of continuing education practices across EU countries highlighted certain patterns and variations in policy, training organisation, and funding mechanisms. The separation between countries with more well-defined national strategies, governmental authorities and stakeholder collaboration, and countries lacking systematic coordination shows the variation and complexity of digital skills development. Countries that showed a stronger emphasis and commitment to the digital skills of HCPs had executed more comprehensive strategies, covering specialised training, workforce development in cooperation with different parties, financial incentives, and mandatory continuing education.

 The findings of this study highlight a fragmented organisation of continuing education within and between countries, posing a risk of uneven quality. The fragmentation is attributed to diverse stakeholders providing education, and the absence of a comprehensive accreditation system exacerbates this issue in many countries. Such a system would serve a dual purpose: ensuring high-quality educational content regardless of the organising entity and providing a structured framework for recognising and awarding credits for the lifelong learning efforts of HCPs. Although some countries have implemented comprehensive accreditation systems, in most countries, only educational institutions providing continuing education are included and employer-provided training omitted.

 The International Medical Informatics Association has established an approach to accredit continuing education programmes aligning with their recommended competences for the use of digital health technologies.^5^ This would not only provide a competitive advantage for educational programmes but also facilitate quality comparisons with global benchmarks and contribute to programme development.^5^ Additionally, the EU framework recognises the opportunities for Member States to exchange best practices and learn from each other in the development of lifelong learning.^14^ Country experiences could also be shared to promote the implementation of comprehensive accreditation systems. HCPs have expressed a desire for their achievements arising from various learning environments to be visible.^15^

 Only a few countries brought up the funding mechanisms for continuing education, as it was not asked of the experts in a separate question. The described funding was diverse, including employer contributions, national authority support, sponsor-based funding, and self-financing by professionals. Shared funding responsibilities within some countries indicates the collaborative nature of supporting the digital skills development of professionals. The continuity of education is susceptible to risks in countries where it is predominantly funded by employers, given the challenging labour market conditions and pressure for cost-cutting measures in healthcare organisations.^16-18^ More shared responsibility for funding would ensure access to continuing education for all HCPs.

###  Limitations

 When interpreting the results of this study, it is important to note that they are primarily based on the perspectives and information provided by individual experts from different countries. The authors were unable to guarantee the accuracy or currency of the information in all respects. It should also be noted that with constantly increasing digitalisation in healthcare, it is likely that continuous change and development will occur in all countries in continuing education. Thus, new practices may have been implemented after the data collection.

## Conclusion

 The study highlights the importance of adopting a systematic approach to enhancing continuous professional development in digital skills, which ensures that professionals have equitable access to education, resulting in consistent, high-quality patient care across countries and regions. Consequently, these findings offer valuable insights for policy-makers, educators, healthcare institutions, and professionals.

## Acknowledgements

 The authors would like to warmly thank all the experts who sacrificed their time and answered the survey. We are also very grateful to those who offered their help to find potential respondents from different countries.

## Ethical issues

 In the research invitation, the respondents were informed that answering the survey was voluntary, that the answers would be anonymised, and that the respondent’s name or organisation would not be disclosed. In addition, a privacy notice was prepared on the processing of personal data, and a link to this was attached to the research invitation. An ethical assessment was considered unnecessary, because the study involved seeking knowledge from experts without an expectation of causing any harm. This decision is in accordance with the regulations set by the Finnish National Board of Research Integrity, which does not mandate an ethical pre-assessment for this type of research in Finland.^14^

## Competing interests

 Authors declare that they have no competing interests.

## Supplementary files

Supplementary file 1. Coding Tree and Country-Specific Information About Continuing Education in Digital Skills for Healthcare Professionals.

Supplementary file 2. Used Online Sources by Country.
