# Supplementary material for: Continuing Education in Digital Skills for Healthcare Professionals — Mapping of the Current Situation in EU Member States
Source: Int J Health Policy Manag. 2024 Jun 24;13:8309. doi: 10.34172/ijhpm.8309 (PMC11365066; doi:10.34172/ijhpm.8309)
Supplement: Supplementary file 1 — Coding Tree and Country-Specific Information About Continuing Education in Digital Skills for Healthcare Professionals. [file ijhpm-13-8309-s001.pdf]

Article title: Continuing Education in Digital Skills for Healthcare Professionals — Mapping of the Current Situation in EU Member States

Journal name: International Journal of Health Policy and Management (IJHPM)

Authors' information: Anu-Marja Kaihlanen[1]¶*, Lotta Virtanen[1]¶, Emma Kainiemi[1], Virpi Sulosaari[2], Tarja Heponiemi[1]

[1]Department of Public Health and Welfare, Finnish Institute for Health and Welfare, Helsinki, Finland.

[2]Faculty of Health and Well-being, Turku University of Applied Sciences, Master School, Turku, Finland

***Correspondence to:** Anu-Marja Kaihlanen; Email: anu.kaihlanen@thl.fi

¶ Both authors contributed equally to this paper.

Citation: Kaihlanen AM, Virtanen L, Kainiemi E, Sulosaari V, Heponiemi T. Continuing education in digital skills for healthcare professionals — mapping of the current situation in EU Member States. Int J Health Policy Manag. 2024;13:8309. doi:10.34172/ijhpm.8309

Supplementary file 1. Coding Tree and Country-Specific Information About Continuing Education in Digital Skills for Healthcare Professionals

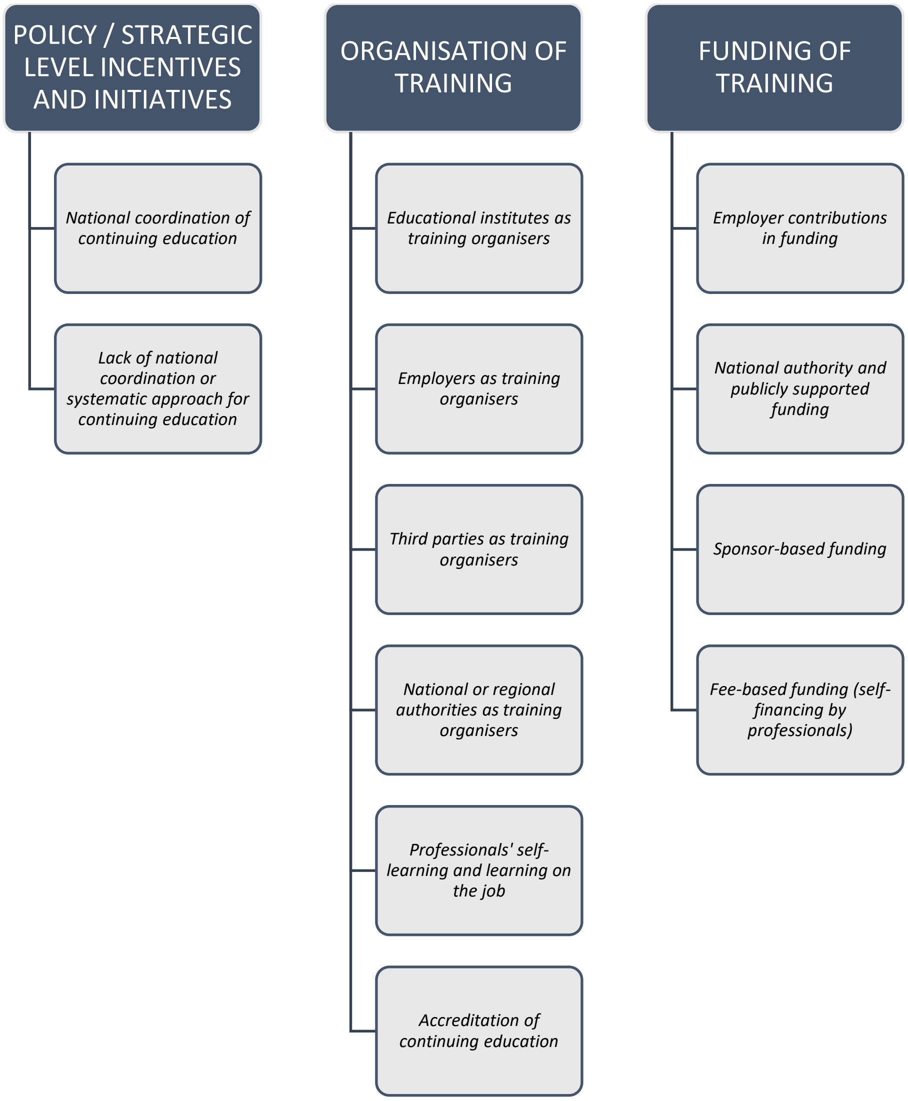

Figure S1: Coding tree of the content analysis. Three main categories and related sub-categories were identified.

Table S1: Country-specific information about incentives and initiatives, organisation, and funding of continuing education in digital skills for healthcare professionals according to data analysis. Main categories are presented in UPPERCASE, sub-categories in *italics*, and condensed expressions from expert quotations or data on documents in quotation marks. n/a means that no data related to that main category was found for the country.

Country	POLICY / STRATEGIC LEVEL INCENTIVES AND INITIATIVES	ORGANISATION OF TRAINING	FUNDING OF TRAINING
Austria	*National coordination of continuing education:*<br>"Continuing education (60 h in 5 years) compulsory for healthcare professionals, monitored via national point system"<br>*Lack of national coordination or systematic approach for continuing education:*<br>"Lack of national coordination in Continuing education in digital skills for healthcare professionals seems to persist despite national goals for population and public sector digital competency"	*Educational institutes as training organisers:*<br>"Fee-based nursing informatics training courses for nurses at universities"<br>"Nurse managers struggle with digitally upskilling staff, as continuing education opportunities are often limited to university with university-level bachelor's degree requirements"<br>"Physicians (and dentists) are offered 2-semesters postgraduate university programme in digital skills"<br>*Employers as training organisers:*<br>"Expert believes large hospitals provide in-house training on digital skills for nurses, e.g., during implementations"<br>*Third parties as training organisers:*<br>"Various third-party stakeholders seem to organise healthcare professionals' accredited continuing education in digital skills"	*Sponsor-based funding:*<br>"Sponsors commonly finance healthcare professionals' continuing training, content independent"<br>*Fee-based funding (self-financing by professionals):*<br>"Fee-based nursing informatics training courses for nurses at universities"
Belgium	*National coordination of continuing education:*<br>"Differences between professions in the obligations imposed on the continuing competence development to maintain a professional license"<br>*Lack of national coordination or systematic approach for continuing education:*<br>"Lack of national regulation for healthcare professionals' continuing education"	*Accreditation of continuing education:*<br>"Currently, no accreditation"	n/a

Bulgaria	n/a	n/a	n/a
Croatia	*National coordination of continuing education:*<br>“The national strategy emphasises the responsibility of universities and trade unions in coordinating professionals’ continuing education “<br>“Based on a national strategy, financial incentives will be established to foster continuing training in general in organisations”	*Accreditation of continuing education:*<br>“Robust accreditation practices for continuing education in digital skills are currently absent, but initiated”	n/a
Republic of Cyprus	*National coordination of continuing education:*<br>“National authorities are responsible for continuing education in digital skills for healthcare professionals”	*Accreditation of continuing education:*<br>“Robust accreditation practices for continuing education in digital skills are currently absent, but initiated”	n/a
Czech Republic	*Lack of national coordination or systematic approach for continuing education:*<br>“Expert is not aware of systematic approach for healthcare professionals’ continuous education in digital skills”	*Educational institutions / Third parties as training organisers:*<br>“There are also non-compulsory courses on the development of digital competences provided at undergraduate level by medical faculties and at postgraduate level by 1. The Institute for Postgraduate Medical Education, 2. National Centre for Nursing and Non-Medical Health Professions and 3. Czech Medical Chamber.”<br>*Employers as training organisers:*<br>“The training of healthcare professionals in digital competences is carried out on an individual level within healthcare institutions, i.e. by healthcare service providers.”	n/a
Denmark	*Lack of national coordination or systematic approach for continuing education:*<br>“No systematic approach or responsibility for developing healthcare professionals’ digital skills”	*National or regional authorities as training organisers:*<br>“Governmental and regional authorities involved in organising Continuing education in digital skills for healthcare professionals”<br>“Regional institute provides simulation post-graduate courses to train healthcare professionals’ digital skills”	n/a

Estonia	*Lack of national coordination or systematic approach for continuing education:*<br>"No systematic national support for Continuing education in digital skills for healthcare professionals"	*Educational institutions as training organisers:*<br>"One university is providing certificated trainings for physicians and health organisations"	n/a
Finland	*National coordination of continuing education:*<br>"Although no centralised management of Continuing education in digital skills for healthcare professionals, recommendations and learning materials are provided at the national level"	*Educational institutions as training organisers:*<br>"One university provides continuing education for physicians' eHealth, mHealth, and telemedicine practice through online courses"<br>"Master's degrees combining healthcare and technology are offered"<br>*Employers as training organisers:*<br>"Healthcare employers are obliged to ensure that their personnel can proficiently use health information systems/devices"<br>"Employers can provide their own continuing education for digital skills"<br>*National or regional authorities as training organisers:*<br>"Learning materials provided at the national level"<br>"National digital service-related portal provides information that healthcare professionals can use to strengthen their digital skills"<br>"During the national implementation of patient data repository, nationwide online training materials were provided"<br>"Employers often allow their employees to complete training related to national digital implementations during working hours"<br>*Third parties as training organisers:*<br>"Third parties sponsored by employers or individuals (professionals?) can provide digital skills proficiency"<br>"Organisations specialising in digital health provide courses for healthcare professionals"<br>"Healthcare professionals can participate in the annual digital health training event"<br>"A scientific and educational institution publishes self-study learning materials and courses online for physicians' continuing education in digital skills"<br>*Accreditation of continuing education:*	*Employer contributions in funding / Fee-based funding (self-financing by professionals):*<br>"Third parties sponsored by employers or individuals (professionals?) can provide digital skills proficiency"

		"Physicians (and dentists and vets) who complete a 2-year additional training in information technology are granted a special competence in healthcare informatics"<br>"Nurses can obtain a special competence in nursing information management"	
France	*National coordination of continuing education:*<br>"Physicians are mandated to take triennial continuous education to maintain licensing, but course selection is optional"<br>"National decree has designated agency to be responsible for overseeing healthcare professionals' continuing education and contributing to its funding"	*Educational institutions as training organisers:*<br>"Universities are obliged to provide continuing training for healthcare professionals"<br>*Accreditation of continuing education:*<br>"Triennial continuous professional development system. The accreditation of such courses depends on the nature of CPD activities undertaken by physicians. Some academic activities may potentially allow participants to earn a certain number of ECTS credits."	*Employer contributions in funding:*<br>"Employers required to contribute to the funding of continuing education for their salaried healthcare professional employees"<br>*National authority and publicly supported funding:*<br>"National authority oversees and contributes to the funding of continuing education for all healthcare professionals"
Germany	*Lack of national coordination or systematic approach for continuing education:*<br>"Despite recommendations, lack of national strategy for Continuing education in digital skills for healthcare professionals"	*Educational institutions as training organisers:*<br>"One university recently started to provide practical digital skills training for healthcare professionals"<br>*Employers as training organisers:*<br>"Some healthcare organisations provide education for digital skills"<br>*Accreditation of continuing education:*<br>"Not applicable"	*National authority and publicly supported funding:*<br>"Federal state funds continuing training programme for healthcare professionals' digital skills"
Greece	*National coordination of continuing education:*<br>"Certification in the basics of digital skills needed for nursing work in the public sector"<br>*Lack of national coordination or systematic approach for continuing education:*<br>"Expert perceives minimal effort in promoting the national strategy for	*Educational institutions as training organisers:*<br>"Universities provide modules for basic digital skills in nursing work"<br>"Educational organisations (e.g., IT or health departments) provide only few continuing education opportunities for an advanced level of digital skills in nursing work"<br>"Some master's programmes related to digital skills are offered"<br>*Accreditation of continuing education:*<br>"Continuing education programmes at universities and master's programmes are accredited"	n/a

	enhancing digital skills among healthcare professionals"<br>"National strategy has not designated specific authority to coordinate the implementation of training"		
Hungary	*National coordination of continuing education:*<br>"Government agency oversees healthcare and its education development in general across all levels"	*Third parties as training organisers:*<br>"National associations for nurses and physicians responsible for supporting, developing, and implementing professionals' continuing education in general"	n/a
Ireland	*Lack of national coordination or systematic approach for continuing education:*<br>"Experts think healthcare professionals' digital skills development is inadequately organised and often depends on the individual professional's interest"	*Educational institutions as training organisers:*<br>"Multiple universities have courses available for continuing education in digital skills for independent development"<br>"Master's programmes in digital health available at multiple universities"<br>*National or regional authorities as training organisers / Professionals' self-learning and learning on the job:*<br>"Professionals can educate themselves on online learning platform provided by national health service authority"<br>*Accreditation of education:*<br>"Primarily, continuing education is accredited at university level in line with Level 9 (postgraduate) of the National Framework for Qualifications"	*Employer contributions in funding / Fee-based funding (self-financing by professionals):*<br>"Employers, or professionals themselves, can fund some of the continuing education provided by third parties"
Italy	*National coordination of continuing education:*<br>"Regional institute coordinates the implementation and evaluation of training activities (in general) of public employees on a regional level"<br>"Recently established accreditation system could be used as a framework for minimum requirements in digital health competencies"	*Educational institutions as training organisers:*<br>"Digital skills training for physicians lies with medial education institutions, but its organisation seems to be insufficient"<br>*Third parties as training organisers:*<br>"Third party organisation coordinates and promotes digital health and its high-level education nationally"<br>*Accreditation of continuing education:*<br>"Robust accreditation practices for continuing education in digital skills are currently absent, but recently suggested an accreditation system could be used as a framework for minimum requirements in digital health competencies"	n/a

	Lack of national coordination or systematic approach for continuing education:<br>"A lack of digital health training for physicians in post-graduate education"<br>"No particular attention on digital skills development but the state has allocated funding for development activities around digitalisation; Digitalisation-related training is in early and uneven stage"		
Latvia	n/a	*Educational institutions / Employers / Third parties as training organisers:*<br>"Continuing education in digital skills is organised by employers, universities and professional associations/organisations"<br>"University training programmes focus on Digital health (managing digital transformation of healthcare) and Digital medicine (managing specific tools needed to digitalise clinical processes)"<br>*Accreditation of continuing education:*<br>"Training courses at university accredited with ECTS credits"	n/a
Lithuania	*Lack of national coordination or systematic approach for continuing education:*<br>"No national coordination yet but under consideration"	*Employers as training organisers:*<br>"Training not much university-based but responsibility lies with each healthcare provider"<br>*National or regional authorities as training organisers:*<br>"Training on the updates and correct use of central national e-health system is organised periodically and on request"<br>*Accreditation of continuing education:*<br>"Currently no official credits dedicated specifically to digital skills, as training is mainly in-house training. However, healthcare professionals can get credits or hours from digital skills trainings with 1/3 rule"	n/a
Luxembourg	n/a	n/a	n/a
Malta	*Lack of national coordination or systematic approach for continuing education:*	*Educational institutions as training organisers:*<br>"New master's programme in Digital Health is expected to catalyse a change and facilitate digital skills development"	n/a

	"Ministry and university as main stakeholders but no main responsible authority coordinating the training"	*Professionals' self-learning and learning on the job:*<br>"Digital skills development mainly self-directed and learned on the job"<br>*Accreditation of continuing education:*<br>"ECTS provided by training at university"	
Netherlands	*National coordination of continuing education:*<br>"Digital skills development is nationally coordinated but voluntary for organisations to offer"<br>"Several parties coordinate the development of digital skills: HR professionals, training professionals from healthcare organisations, the IT department and the digital skills project manager"<br>"Minimum digital skills needed by professionals in different healthcare sectors are defined at national level"<br>*Lack of national coordination or systematic approach for continuing education:*<br>"After basic education professionals' digital skills are not at a sufficient level: Insufficient integration of digital skills into basic education/curricula"	*National or regional authorities as training organisers:*<br>"National programme funded by the government provides a website with free online learning materials and arranges events that are available for healthcare organisations and professionals to assist in digital skills"<br>*Third parties as training organisers:*<br>"A private company trains healthcare professionals as digital coaches who can act as supporters of other professionals in their organisations"<br>*Accreditation of education:*<br>"The training of the trainers is accredited. Self-learning trainings not accredited"	*Fee-based funding (self-financing by professionals):*<br>"Physicians often finance their own continuing education"
Poland	*National coordination of continuing education:*<br>"National programme aims to introduce a qualification system to outline digital skills healthcare professionals' need"<br>"National institute has been established to coordinate professionals' digital skills development in collaboration with various stakeholders"	*Third parties as training organisers:*<br>"Nursing organisations, in cooperation with nursing leaders and the International Council of Nurses, organise life-long learning courses in general for nurses"<br>*Accreditation of education:*<br>"Robust accreditation practices for continuing education in digital skills are currently absent, but initiated"	n/a

Portugal	*Lack of national coordination or systematic approach for continuing education:*<br>"Specific coordination of healthcare professionals' national strategy seems to be lacking, although strategy exists to foster employees' digital skills at the population level"	*Educational institutions as training organisers:*<br>"Postgraduate university organise accredited international postgraduate digital skills course for healthcare professionals in a hybrid format"	n/a
Romania	*Lack of national coordination or systematic approach for continuing education:*<br>"No national authority coordinating digital skills development"	*Educational institutions as training organisers:*<br>"Courses/training can be organised locally as a part of continuing medical education in universities or hospitals"<br>*Employers as training organisers:*<br>"No government-run programmes but development of skills is organised on an institutional/organisational level"<br>*Accreditation of continuing education:*<br>"Professional governing bodies can accredit with CME credits trainings, workshops, and courses in general"	n/a
Slovakia	*National coordination of continuing education:*<br>"Healthcare Supervision Office as a main coordinator of implementing training / Training coordinated by governmental agency"<br>"Development of digital skills is included in a specific continuing education programme"	*Accreditation of continuing education:*<br>"Continuing education is accredited, leading to a certificate"	n/a
Slovenia	*National coordination of continuing education:*<br>"Physicians are mandated to obtain continuing medical education accreditations or take a re-certification exam to maintain their license every 7 years (until age 70)"<br>"Ensuring healthcare professionals' specialisation in digitalisation is a	*Employers as training organisers:*<br>"Continuing training seems to be dependent on organisational practices"<br>*Third parties as training organisers:*<br>"Professional associations have a central position in organising continuing training in general"<br>"Training often limited to learning by doing with peer support"<br>*Professionals' self-learning and learning on the job:*	n/a

	national strategic goal (e.g., through state-supported funding), but has not yet been implemented in practice"<br>"Centralised competency units will be established to be responsible for healthcare professionals' continuing training in digital skills and training the new profession that combines IT and healthcare expertise"	"Self-learning emerges as prevalent for healthcare professionals to develop digital skills"<br>*Accreditation of continuing education:*<br>"Robust accreditation practices for continuing education in digital skills are currently absent, but initiated"	
Spain	*National coordination of continuing education:*<br>"Ongoing national initiative 'Digital Health Strategy' includes planning of a specialised digital training for professionals"<br>"Ministry sets guidelines for training but is not involved in implementing/coordinating of training or defining competencies"<br>"Training is coordinated by regional governments (not by national authority) thus level of implementation varies, depending on local priorities and emphasis"	*Educational institutions / Employers / Third parties / National or regional authorities as training organisers:*<br>"Training is organised by universities, associations (professional, public and private), health departments and national and local governments"<br>*Accreditation of continuing education:*<br>"Robust accreditation practices for continuing education in digital skills are currently absent, but initiated"	n/a
Sweden	*Lack of national coordination or systematic approach for continuing education:*<br>"Expert is not aware of any national authority responsible for coordinating continuing training in digital skills"	*Educational institutions as training organisers:*<br>"Healthcare professionals are offered continuing education in digital skills by courses in higher education at universities"<br>*Employers as training organisers:*<br>"The employers are at some extent organising in-house training in digital skills"<br>*Accreditation of continuing education:*<br>"Continuing education at universities is accredited with ECTS"<br>"In-house training was stated to be possibly resulting in some kind of certificate at a local level"	n/a
