# Supplementary material for: Continuing Education in Digital Skills for Healthcare Professionals — Mapping of the Current Situation in EU Member States
Source: Int J Health Policy Manag. 2024 Jun 24;13:8309. doi: 10.34172/ijhpm.8309 (PMC11365066; doi:10.34172/ijhpm.8309)
Supplement: Supplementary file 2 — Used Online Sources by Country. [file ijhpm-13-8309-s002.pdf]

Article title: Continuing Education in Digital Skills for Healthcare Professionals — Mapping of the Current Situation in EU Member States

Journal name: International Journal of Health Policy and Management (IJHPM)

Authors' information: Anu-Marja Kaihlanen[1]¶*, Lotta Virtanen[1]¶, Emma Kainiemi[1], Virpi Sulosaari[2], Tarja Heponiemi[1]

[1]Department of Public Health and Welfare, Finnish Institute for Health and Welfare, Helsinki, Finland.

[2]Faculty of Health and Well-being, Turku University of Applied Sciences, Master School, Turku, Finland

***Correspondence to:** Anu-Marja Kaihlanen; Email: anu.kaihlanen@thl.fi

¶ Both authors contributed equally to this paper.

Citation: Kaihlanen AM, Virtanen L, Kainiemi E, Sulosaari V, Heponiemi T. Continuing education in digital skills for healthcare professionals — mapping of the current situation in EU Member States. Int J Health Policy Manag. 2024;13:8309. doi:10.34172/ijhpm.8309

Supplementary file 2. Used Online Sources by Country

Austria

Ammenwerth, E., & Hackl, W. O. (2019). Topics for Continuous Education in Nursing Informatics: Results of a Survey Among 280 Austrian Nurses. Studies in health technology and informatics, 260, 162–169. https://doi.org/10.3233/978-1-61499-971-3-162

Digital Austria. (2023). Digitale Kompetenzoffensive für Österreich. https://www.digitalaustria.gv.at/Strategien/DKO-Digitale-Kompetenzoffensive.html

Egbert, N., Thye, J., Hackl, W. O., Müller-Staub, M., Ammenwerth, E., & Hübner, U. (2019). Competencies for nursing in a digital world. Methodology, results, and use of the DACH-recommendations for nursing informatics core competency areas in Austria, Germany, and

Switzerland. Informatics for health & social care, 44(4), 351–375. https://doi.org/10.1080/17538157.2018.1497635

Hassler, M. (2022). Digitalization and new technologies in care – concepts and potentials for nursing care provision. In: Primic, N (Ed.). Digitalisierung der Pflege. Interdisziplinäre Perspektiven auf digitale Transformation in der pflegerischen Praxis. V&R unipress.

Medizinische Universität Graz. (2023). Erweiterungsstudium digitalisierung in der medizin. https://www.medunigraz.at/erweiterungsstudien/digitalisierung-in-der-medizin

Rieder, A. (2020). Ausbildung zukünftiger Ärztinnen und Ärzte im Zusammenhang mit der digitalen Transformation und Covid-19 als Herausforderer im Medizinstudium. In F. Wallner & P. Niedermoser (Eds.), Schwerpunktthema Telemedizin – Corona als Wendepunkt (pp. 61–78). Ausgabe 2/2020. LIG – Linzer Institut für Gesundheitssystem-Forschung. https://www.telemedaustria.at/images/uploads/2020_Telemedizin_oe.pdf

Telemed Austria. (2022). Telemed Austria joins the ISfTeH. https://www.telemedaustria.at/news/97-telemed-austria-joins-the-isfteh

Viegelsundheit. (n.d.). FAQ - Häufige Fragen und Hilfe. https://www.vielgesundheit.at/fortbildungen/faq

Österreichischen Ärztekammer. (2020). Verordnung über ärztliche fortbildung. https://www.arztakademie.at/diplom-fortbildungs-programm/grundsaetzliches-begriffe/verordnung-ueber-aerztliche-fortbildung/

Croatia

Ministry of Science and Education. (2017). New Colours of Knowledge. Strategy for education, science and technology. http://www.kvalifikacije.hr/sites/default/files/documents-publications/2018-03/SOZT_engleski.pdf

Denmark

Copenhagen Academy for Medical Education and Simulation. (n.d). Simulation courses and training sessions education. https://cames.dk/en/uddannelse-og-traening/#digital|1||kursusoversigt|0

Germany

Macher, J. (2023). Neue Weiterbildung für digitale Pflege- und Gesundheitsversorgung: Kostenfreie Teilnahme an Modellprojekt möglich. Informationsdienst Wissenschaft. https://idw-online.de/de/news808656

Hungary

European Union. (n.d). Digital skills & jobs platform. Hungarian Ministry of Human Resources. https://digital-skills-jobs.europa.eu/en/organisations/hungarian-ministry-human-resources

Magyar Egészségügyi Szakdolgozói Kamara – MESZK. (2023). Jelenlegi MESZK E-Továbbképzések. https://meszk.hu/cikk/2023/06/08/jelenlegi-meszk-e-tovabbkepzesek

Magyar Orvosi Kamara – MOK. (2023). A Magyar Orvosi Kamara Missziós Nyilatkozata. https://mok.hu/a-kamararol/kuldetesunk-alapelveink

Italy

Casà, C., Marotta, C., Di Pumpo, M., Cozzolino, A., D'Aviero, A., Frisicale, E. M., Silenzi, A., Gabbrielli, F., Bertinato, L., & Brusaferro, S. (2021). COVID-19 and digital competencies among young physicians: are we (really) ready for the new era? A national survey of the Italian Young Medical Doctors Association. Annali dell'Istituto superiore di sanita, 57(1), 1–6. https://doi.org/10.4415/ANN_21_01_01

Marceglia, S., Balestra, G., Bottrighi, A., Giacomini, M., Veltri, P., & Sacchi, L. (2022). Developing the Digital Healthcare Workforce in Italy: The SIBIM Experience. Studies in health technology and informatics, 298, 46–50. https://doi.org/10.3233/SHTI220905

Netherlands

Löffler, Christin, Altiner, Attila, Blumenthal, Sandra, Bruno, Pascale, De Sutter, An, De Vos, Bart J., Dinant, Geert-Jan, Duerden, Martin, Dunais, Brigitte, Egidi, Günther, Gibis, Bernhard, Melbye, Hasse, Rouquier, Frederic, Rosemann, Thomas, Touboul-Lundgren, Pia, & Feldmeier, Gregor. (2022). Challenges and opportunities for general practice specific CME in Europe – a narrative review of seven countries. BMC Medical Education, 22(1), 761. https://doi.org/10.1186/s12909-022-03832-7

Poland

Bartosiewicz, A., Burzyńska, J., & Januszewicz, P. (2021). Polish Nurses' Attitude to e-Health Solutions and Self-Assessment of Their IT Competence. Journal of Clinical Medicine, 10, 4799. https://doi.org/10.3390/jcm10204799

Burzyńska, J., Bartosiewicz, A., & Januszewicz, P. (2023). Dr. Google: Physicians-The Web-Patients Triangle: Digital Skills and Attitudes towards e-Health Solutions among Physicians in South Eastern Poland-A Cross-Sectional Study in a Pre-COVID-19 Era. International journal of environmental research and public health, 20(2), 978. https://doi.org/10.3390/ijerph20020978

Dębska, G., Gorzkowicz, B., Foryś, Z., & Kilańska, D. (2020). Continuous professional development of nurses and ICNP® introduction in Poland. International journal of occupational medicine and environmental health, 33(3), 353–363. https://doi.org/10.13075/ijomeh.1896.01480

Jākobsone, M. (2021). Poland - Digital Competence Development Programme (2020-2030). https://digital-skills-jobs.europa.eu/en/actions/national-initiatives/national-strategies/poland-digital-competence-development-programme

Ministerstwo Cyfryzacji. (2023). Kompetencje cyfrowe. https://www.gov.pl/web/cyfryzacja/kompetencje-cyfrowe

Staelraeve, S. (2023). Poland is an emerging market for telehealth in Europe. https://www.dashplus.be/business-advice/poland-is-an-emerging-market-for-telehealth-in-europe/

Portugal

National School of Public Health, Nova University Lisbon. (n.d). Postgraduate courses: Digital Health Executive Course. https://www.ensp.unl.pt/courses/postgraduate-courses/digital-health-executive-course/

ReferNet Portugal, Cedefop. (2023). Strengthening digital skills for competitiveness and inclusion. National news on VET. https://www.cedefop.europa.eu/fr/news/portugal-strengthening-digital-skills-competitiveness-and-inclusion

Slovenia

Boesen, R. L., Frydensberg, M. S., & Justi, L. (2022). Digital educational programme invoiving health professionals (DELIVER): Research report intellectual output 1: Need analysis. https://project-deliver.eu/wp-content/uploads/2022/09/DELIVER-report-IO1_FINAL.pdf

Republika Slovenija Ministrstvo Za Zdravje. (2022). Slovenija – e-zdravje za bolj zdravo družbo(REFORM/SC2021/061). Republika Slovenija Ministrstvo Za Zdravje. https://www.gov.si/assets/ministrstva/MZ/DOKUMENTI/O-MINISTRSTVU/Slovenija-E-zdravje-za-bolj-zdravo-druzbo-v2.pdf
